# Gli as a Novel Therapeutic Target in Malignant Pleural Mesothelioma

**DOI:** 10.1371/journal.pone.0057346

**Published:** 2013-03-06

**Authors:** Hui Li, Natalie Lui, Tiffany Cheng, Hsin-Hui K. Tseng, Dongsheng Yue, Etienne Giroux-Leprieur, Hanh T. Do, Qing Sheng, Joy Q. Jin, Thomas W. Luh, David M. Jablons, Biao He

**Affiliations:** 1 Thoracic Oncology Program, Department of Surgery, University of California San Francisco, San Francisco, California, United States of America; 2 College of Life Sciences, Zhejiang Sci-Tech University, Hangzhou, P.R.China; 3 Department of Lung Cancer, Lung Cancer Center, TianJin Medical University Cancer Institute and Hospital, Tianjin, P.R.China; Indiana University School of Medicine, United States of America

## Abstract

Malignant pleural mesothelioma (MPM) is a highly aggressive tumor with poor prognosis. Current treatment is rarely curative, thus novel meaningful therapies are urgently needed. Inhibition of Hedgehog (Hh) signaling at the cell membrane level in several cancers has shown anti-cancer activity in recent clinical studies. Evidence of Hh-independent Gli activation suggests Gli as a more potent therapeutic target. The current study is aimed to evaluate the potential of Gli as a therapeutic target to treat MPM. The expression profiles of Gli factors and other Hh signaling components were characterized in 46 MPM patient tissue samples by RT-PCR and immunohistochemistry. Cultured cell lines were employed to investigate the requirement of Gli activation in tumor cell growth by inhibiting Gli through siRNA or a novel small molecule Gli inhibitor (Gli-I). A xenograft model was used to evaluate Gli-I *in vivo*. In addition, a side by side comparison between Gli and Smoothened (Smo) inhibition was conducted *in vitro* using siRNA and small molecule inhibitors. Our study reported aberrant Gli1 and Gli2 activation in a large majority of tissues. Inhibition of Gli by siRNAs or Gli-I suppressed cell growth dramatically both *in vitro* and *in vivo*. Inhibition of Gli exhibited better cytotoxicity than that of Smo by siRNA and small molecule inhibitors vismodegib and cyclopamine. Combination of Gli-I and pemetrexed, as well as Gli-I and vismodegib demonstrated synergistic effects in suppression of MPM proliferation *in vitro*. In summary, Gli activation plays a critical role in MPM. Inhibition of Gli function holds strong potential to become a novel, clinically effective approach to treat MPM.

## Introduction

Malignant pleura mesothelioma (MPM) is an uncommon but inexorably fatal cancer that arises from the surface serosal cells of the pleura and, less frequently, from the peritoneum [1]–[3]. Treatment of MPM with surgery, chemotherapy, or radiation therapy is rarely curative with a median survival ranging from 10 to17 months [2]. Despite some promising results, long-term survival with currently available treatment is rare [3], [4]. Therefore, novel meaningful therapies for MPM are urgently needed.

Currently, in spite of frequent observation of NF-kB, EGFR, and PI3K/AKT signaling deregulation in MPM cells, the molecular mechanism underlying tumorigenesis in MPM is poorly understood [1], [5]–[7]. The Hedgehog (Hh) signaling pathway has been implicated in a wide variety of cancers, including leukemia, lung, brain, skin, head and neck, liver, gastrointestinal, colorectal, pancreatic, prostate, mammary, ovarian and renal carcinomas[8]–[12]. Therefore, exploring the role of the Hh pathway in MPM and inhibiting its aberrant activation holds great promise to provide novel and effective treatments for MPM patients.

In the quiescent state of the Hh pathway, the twelve-pass trans-membrane receptor Patched-1 (Ptch1) restrains the activity of the seven-pass trans-membrane receptor Smoothened (Smo) [10], [12]. Binding of Hh ligands to Ptch1 reverses the inhibitory effect on Smo. Activated Smo elicits a complex series of cytoplasmic signal transduction events resulting in activation of the Glioma-associated oncogene (Gli) family of transcription factors. The Gli transcription factors then translate the extra-cellular Hh-stimulus into defined transcriptional programs in a context-dependent and cell-type specific manner [10], [12].

The aberrant activation of Hh signaling happens at several levels throughout the pathway, contributing to the development of many aggressive and metastatic cancers [12]. Conventionally, the frequent activation of the Hh pathway in tumors is thought to be mainly due to overexpression of ligands, loss of Ptch or constitutive active mutants of Smo [8], [10], [12]. Most efforts have been devoted to investigate the inhibition at the cell membrane level, i.e. Smo and Hh inhibitors [12]. The most clinically advanced example is vismodegib (also known as GDC-0449), which was newly approved by the U.S. Food and Drug Administration to treat adult patients with basal cell carcinoma [13]–[15]. Multiple clinical trials are evaluating the use of vismodegib in other types of cancer, as well as several other candidate drugs that target Hh signaling [12], [15].

Downstream Hh pathway activation has also been documented in tumors of the brain, prostate, muscle and in cell lines derived from pancreatic and lung cancers [9], [16]–[21]. The attributed molecular mechanism includes loss of other Hh pathway factors downstream of Hh/Smo and upstream of Gli, such as Sufu and Ren, and Gli gene amplification and chromosomal translocation. Furthermore, a growing body of evidence has revealed additional mechanisms of Gli activation which are independent of Hh/Smo regulation [22]. The Hh-independent Gli activation is stimulated by cross-talk between components downstream of Hh/Smo and several other oncogenic signaling pathways, such as the transforming growth factor β (TGFβ), epidermal growth factor receptor (EGFR), RAS and AKT/PI3K pathways [8], [23]–[32]. Overall, the concept that Gli proteins serve as an integration point of several signaling cascades, in addition to canonical activation from Hh/Smo, has significant implications for the understanding of tumor development. It strongly argues for the strategy to develop novel therapies that target Gli proteins in order to treat aggressive tumors, such as MPM.

The current study investigated the aberrant activation of Gli proteints in MPM, explored the effectiveness of targeted inhibition by a novel Gli inhibitor (Gli-I) to inhibit MPM cell growth, and compared the efficacy of Smo and Gli inhibitors. Our result strongly suggests that targeting Gli factors holds strong potential to become clinically effective treatment options for MPM patients in the near future.

## Materials and Methods

### Ethics Statement

The study with patient tissues was approved by the Committee on Human Research (CHR approval number: H8714-11647-10) at the University of California, San Francisco (UCSF). Written, informed consent was obtained from each patient before specimen collection. Mice study was carried out in strict accordance with the recommendations in the Guide for the Care and Use of Laboratory Animals of the National Institutes of Health. The protocol was approved by the Office of Ethics and Compliance of UCSF.

### Patient Tissues

Tissue specimens were collected from 46 patients who underwent surgical resection for MPM at the Thoracic Oncology Program at UCSF. Samples were frozen immediately and stored in liquid nitrogen until use. Twenty-seven samples were fixed in formalin and embedded in paraffin to make tissue slides.

### Immunohistochemistry, Immunofluorescence and Western Blot

Immunohistochemistry, immunofluorescence and western blot were performed following standard procedures. Antibodies applied to detect protein expressions were Gli1(Santa Cruz Biotechnology, Santa Cruz, CA), Gli2(Abcam, UK), SHh(Abcam), Smo (Sigma, St. Louis, MO), Ki67(Cell Signaling, Beverly, MA), active Caspase 3 (Cell Signaling) and Actin(Sigma). Total protein extraction was performed with M-PER Mammalian Protein Extraction Solution (Thermo Scientific, Waltham, MA), and 40 ug of proteins were analyzed in western blot.

### RNA Extraction and RT-PCR

Total RNA was isolated from tissue or cultured cells using a RNeasy kit (Qiagen, Germany). Genomic DNA contamination was eliminated by DNase I treatment. Reverse transcription was conducted with 250 ng RNA using an iScript cDNA synthesis kit (Bio-Rad, Herculas, CA). The resulting cDNAs were analyzed with real-time RT-PCR using Gene Expression Assays in a 7900 Real-Time PCR System (Applied Biosystems, Foster City, CA) for 40 cycles (96°C for 15 seconds and 60°C for 1 minute). Gene expression was normalized to 18S expression. We defined the Ct value of negative controls (RT minus controls) as the baseline to calculate relative mRNA expression.

### Cell Culture, Drug Treatment

Human mesothelioma cell lines NCI-H28, MS-1, REN, H2052, H2452 and H290 were purchased from the Cell Culture Core Facility at Harvard University (Boston, MA,USA). The cell lines were cultured in RPMI 1640 (Life Technologies, Carlsbad, CA) supplemented with 10% fetal bovine serum (FBS) and antibiotics. Cells were seeded one day before treatment with Gli-I, cyclopamine (Selleck Chemicals) and vismodegib (Selleck) at different concentrations for 30, 48 or 72 hours, with vehicle (DMSO) as controls. Cells were subjected to the following analyses of immunofluorescence, RNA extraction and RT-PCR, TUNEL or proliferation assays.

### Proliferation Assays and siRNA Transfection

Cells were treated with Gli-I, cyclopamine or vismodegib or transfected with siRNAs. Cell proliferation was monitored for 4 days with CellTiterGlo assay (Promega). Proliferation assays were performed for at least three times, and representative results were illustrated. Cells were transient transfected using Lipofectamine 2000 (Life Technology) with siRNAs at a total concentration of 50 nmol/L. In double siRNA treatments, the total siRNA concentration was the same as single siRNA treatments. All siRNAs were purchased from Life Technologies. The efficiency of siRNAs was evaluated by western blot.

### TUNEL Assay

TUNEL assay was performed using the DeadEnd Flurometric TUNEL System (Promega) per standard protocol for both FFPE tissue sections and culture cells. REN, MS1 and H28 were treated with either Gli-I or vismodegib for 40 hr before TUNEL assays. Each experiment was performed for three times.

### Mice Study

Nude mice were subcutaneously injected with 10 million MS-1 cells with BD Matrigel Matrix (BD Biosciences, San Jose, CA). Fourteen days after inoculation, mice were randomized to intraperitoneal injections of either Gli-I at 50 mg/kg or vehicle alone for 14 consecutive days followed by another 7-day observation. Tumor volumes were measured from day 10 to day 35 post-inoculation. Tumors and organs were dissected on day 35 to make paraffin blocks for follow-up analyses. All experiments were performed at the UCSF Preclinical Core.

### Statistical Analysis and Combination Index Analysis

Two-sided student’s t-test was performed for proliferation assays and mRNA expression analysis. A p value <0.05 was indicated as *, 0.01 as **, and 0.001 as *** in corresponding figures. The combinational effects were quantified using the Chou-Talalay Method to obtain the Combinational Index (CI), where CI <1, = 1, >1 represent synergism, additive effect, and antagonism respectively.

## Results

### Gli Family of Transcriptional Factors are Expressed in Malignant Pleural Mesothelioma

We first investigated the gene expression of Gli family of transcriptional factors as well as key SHh pathway components in MPM patient tissues. Tissue specimens were collected from 46 patients who underwent surgical resection for MPM at the Thoracic Oncology Program at the University of California, San Francisco. Among all patients, 11 patients were female, and 35 were male, with an average (± standard deviation) age of 67.2±10.7 years. Histologic analysis showed 39 (85%) epithelioid, 2 (4%) sarcomatous, and 5 (11%) undetermined tumors. There were 5 (11%), 8 (17%), 11 (24%) and 3 (7%) tumors at stage I, II, III and IV respectively, with 19 (41%) undetermined. Tumor samples were collected from all 46 patients, and normal adjacent pleura samples were available from 7 patients. Formalin-fixed, paraffin-embedded tissues were available from 27 patients.

**Figure 1:**
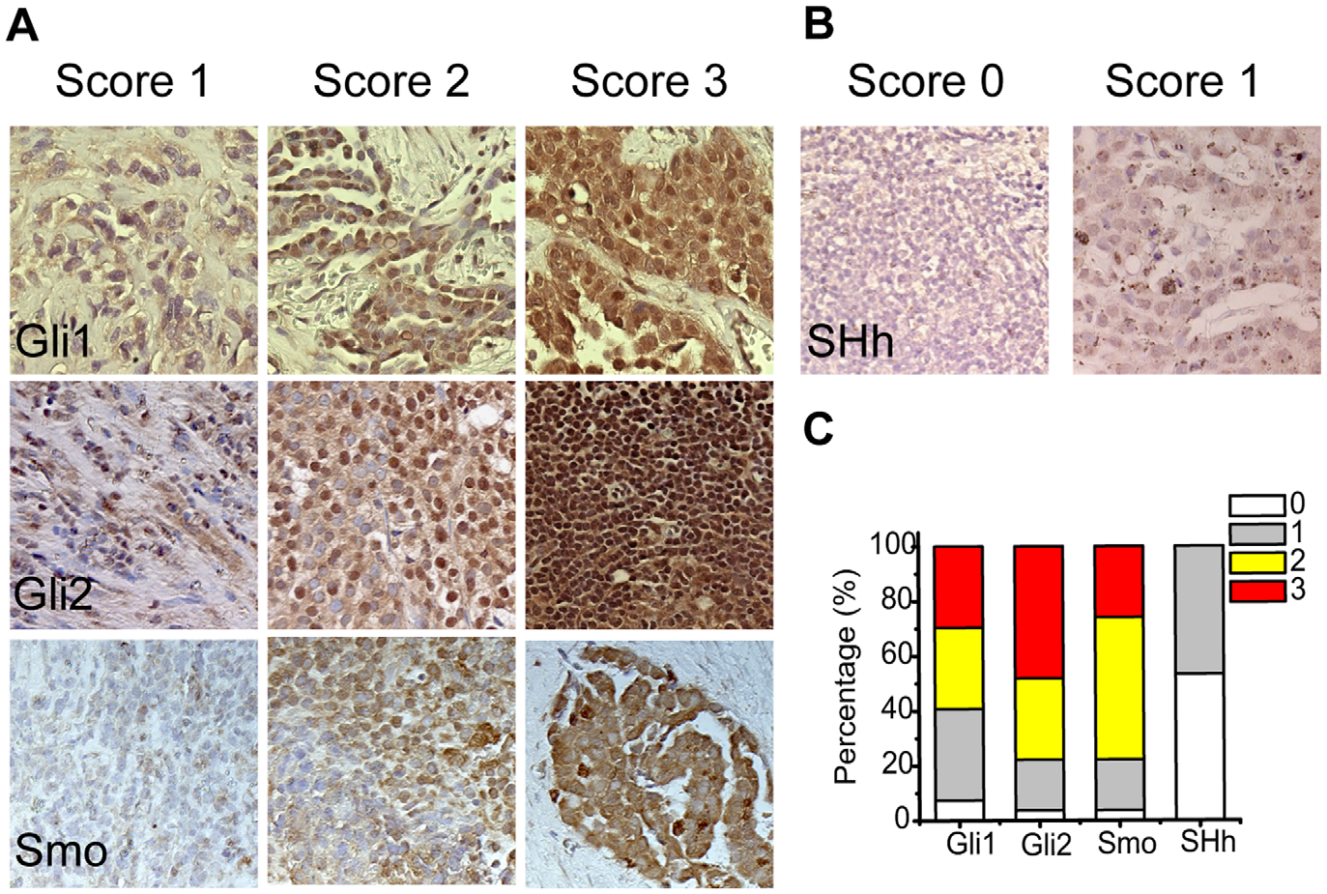
Protein Expression of Gli and SHh Pathway Components in MPM. A , Representative protein expression of Gli1(first row), Gli2(second row) and Smo (third row). Immunohistochemistry staining was scored as 0–3. Representative images of score 1 (first column), 2 (second column), and 3 (third column) were shown. **B**, Representative SHh protein expression. Representative images of score 0 (left panel) and score 1(right panel) were shown. **C**, Expression profiles of Gli1, Gli2, Smo, SHh in MPM tissues. Percentage of score 0–3 of Gli1, Gli2, Smo and SHh was summarized.

**Figure 2:**
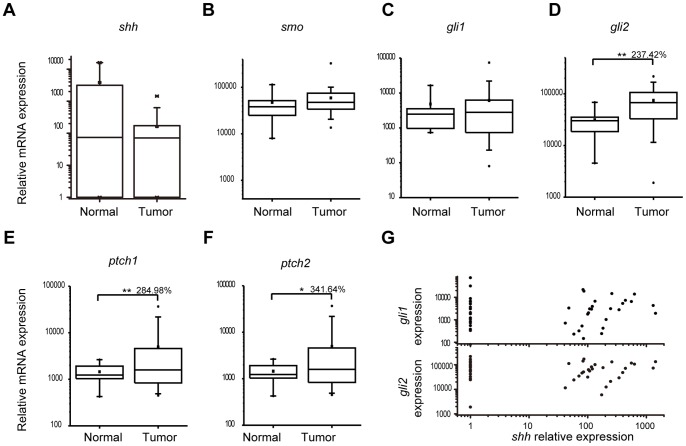
RNA Expression of Gli and SHh Pathway Components in MPM. A–F , Statistic analysis of *shh, smo, gli1, gli2, ptch1* and *patch2* mRNA expression in normal pleura and tumor tissues. A total of 46 tumor tissues was compared with 7 patient normal pleura for its relative mRNA levels of *shh, smo, gli1, gli2, ptch1, ptch2* by quantitative RT-PCR. Two-sided student’s t-test was performed between normal and tumor tissues. *gli2, ptch1* and *ptch2* showed significant elevation (D–F). A p value <0.05 was indicated as *, <0.01 as **, and <0.001 as ***. The average expression of tumor tissues was labeled in the three genes with the average of normal pleura as 100% (D–F). **G,** Correlation analysis between *shh* and *gli1* (upper) and *gli2* (lower). The expression of *shh* was undetectable in 43% of samples, and thereby shown as the baseline level.

**Figure 3:**
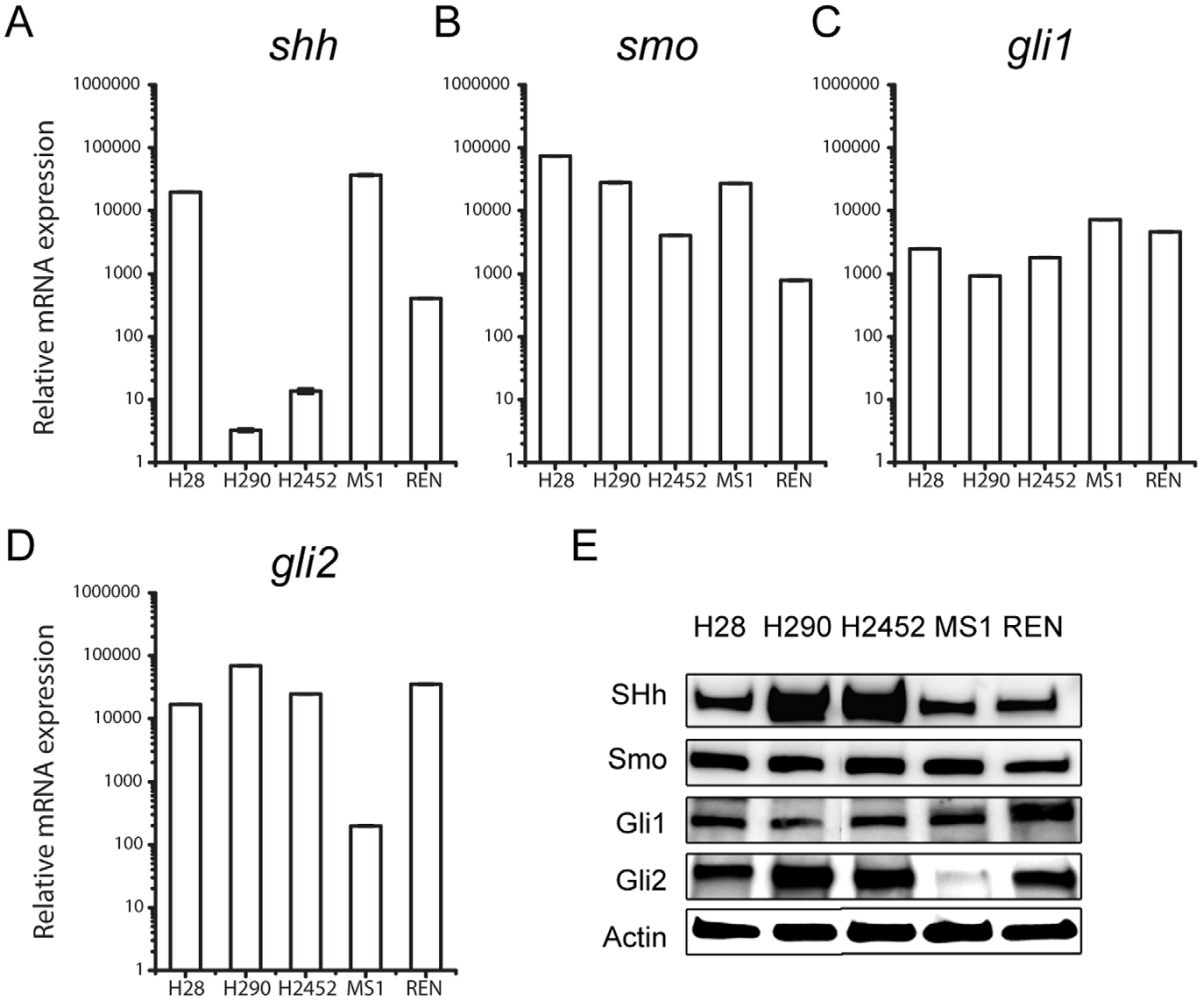
Expression of Gli and SHh Pathway Components in MPM Cell Lines. A–D , Expression of *shh* (A), *smo* (B), *gli1* (C) and *gli2* (D) by quantitative RT-PCR. **E**, Protein expression of SHh, Smo, Gli1 and Gli2.

The protein expression of Gli1, Gli2, Smo and SHh were characterized by immunohistocehmistry (IHC), and scored on a scale of 0–3 (negative, mild, moderate and strong positive). Representative samples in each category of the four proteins were summarized in Figure 1 A. More than 90% of the samples were positive for Gli1, Gli2 and Smo, and a majority of them had moderate to strong expression (Fig. 1C). In contrast, only 47% of the samples exhibited mild SHh expression (Fig. 1B, C). The expression profiles were further characterized by quantitative RT-PCR (qPCR). The expression of 46 tumor samples was compared with that of 7 normal pleura for *shh*, *smo*, *gli1*, *gli2*, *ptch1* and *ptch2* (Fig. 2A–F). The average expression level of *gli2* was 2.4 fold as high in tumor tissues as in patient normal pleura (p<0.01), whereas *gli1* levels were statistically comparable (Fig. 2C, D). Consistently, Gli target genes *ptch1* and *ptch2* were 2.8 (p<0.01) and 3.4 (p<0.05) fold as high as in tumor tissues respectively (Fig. 2E, F). SHh, Smo, Gli1 and Gli2 were expressed in five tested MPM cell lines (Fig. 3). Strong expression of Gli factors and elevated expression of their target genes in tissues indicated that the aberrant Gli activation may play an active role in MPM.

Interestingly, the expression of Gli factors was poorly correlated with their upstream stimulus SHh. Only 47% of the samples showed mild SHh expression, in contrast to more than 90% for Gli1 and Gli2 (Fig. 1C). At mRNA levels, *shh* was detectable in only 57% of the tumor samples, but also in 3 patient normal controls (Fig. 2A). When *shh* was plotted with *gli1* and *gli2* expression, no correlation was observed (Fig. 2G). No correlation between *smo* and *gli1* or *gli2* was observed either (Fig. S1). The lack of correlation was also confirmed in cultured MPM cells. No correlation was obvious between *shh* and its downstream factors *gli1* and *gli2* (Fig. 3A–D) at mRNA levels or at protein levels (Fig. 3E). Aberrant Gli1 activation and the lack of correlation with the upstream SHh signals in MPM prompted us to investigate the oncogenic role of Gli transcriptional factors.

### Downregulation of Gli1 and Gli2 Inhibited Cell Proliferation in Malignant Pleural Mesothelioma Cell Lines

**Figure 4:**
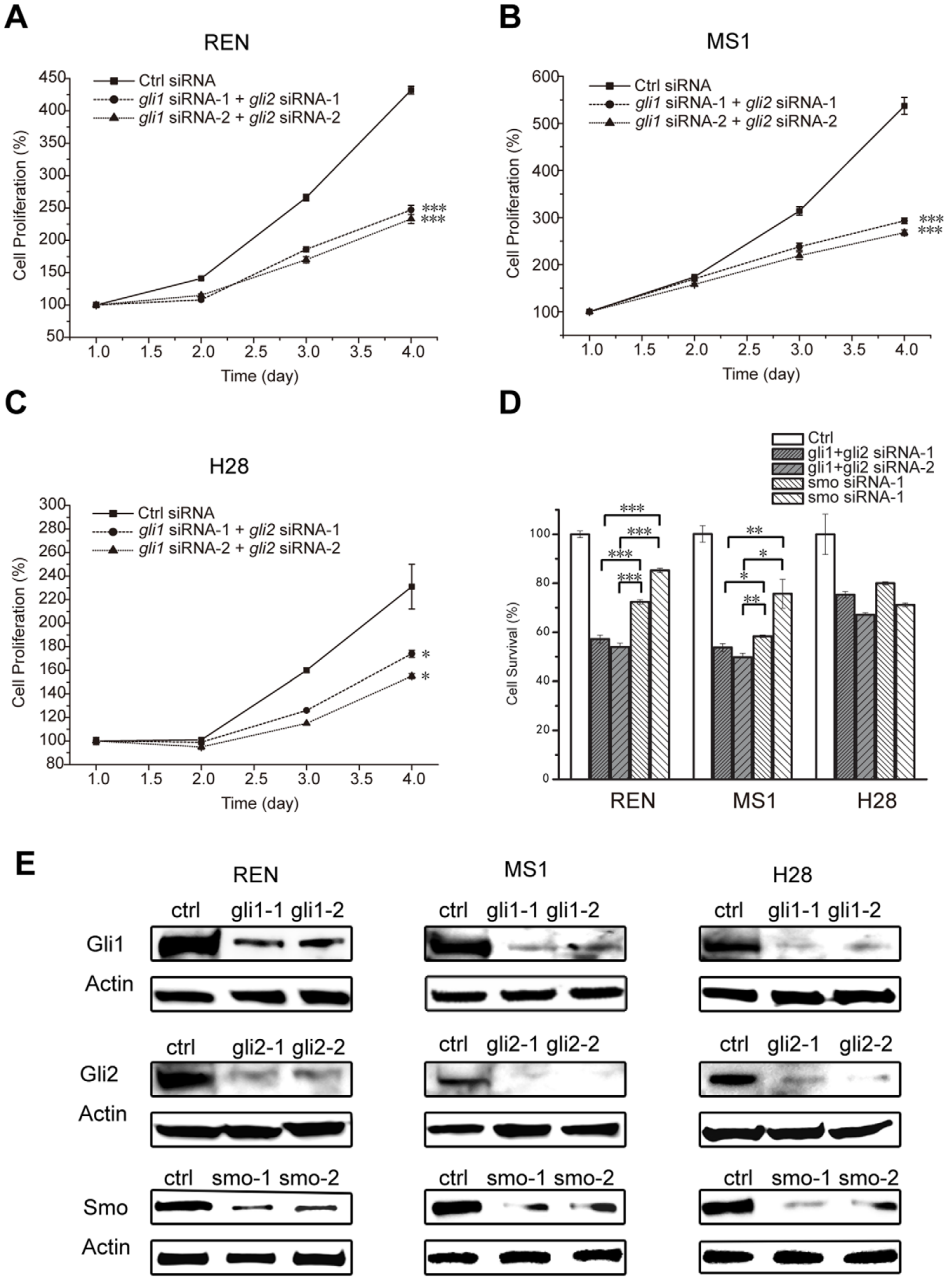
Suppression of Gli1 and Gli2 by siRNA Inhibited Cell Proliferation. A-C , Cell proliferation was significantly reduced by double siRNA treatment targeting *gli1* and *gli2* in three MPM cell lines. Two sets of independent siRNA were applied in each assay. MTS assay was used to measure cell proliferation at 72 hours after siRNA transfection. **D**, Comparison of Smo and Gli inhibition at suppressing cell proliferation at 72 hours after siRNA transfection. Two-sided student’s t-test was performed between control siRNA and *gli* siRNA (A-C), between *gli* siRNA and *smo* siRNA in REN and MS1 (D). A p value <0.05 was indicated as *, <0.01 as **, and <0.001 as ***. **E**, The efficiency of siRNA was monitored by western blot at 72 hours after transfection in REN MS1 H28. Each gene was knocked down with two independent siRNAs.

To investigate the tumorigenic function of Gli factors, we down-regulated Gli1 and Gli2 simultaneously to measure cell proliferation in MPM cell lines. We selected three cell lines to represent different expression profiles of key SHh signaling components: H28 had relatively high expression of Smo and SHh and low expression of Gli factors; whereas REN and MS-1 had the opposite expression profiles (Fig. 3). We suspected that H28 might harbor a dominant upstream SHh signaling, whereas REN and MS-1 might not. Suppression of Gli1 and Gli2 by two sets of independent validated siRNAs (Fig. 4E) resulted in significant inhibition of cell proliferation in 72 hours in all three cell lines (Fig. 4A–C), suggesting the tumorigenic role of Gli factors in MPM. Single treatment of *gli1* or *gli2* siRNA did not show significant inhibitory effects of cell proliferation (data not shown), which might be explained by the redundancy of the two transcriptional factors. The application of *gli1* and *gli2* siRNAs resulted in significantly better inhibition of cell survival than *smo* siRNAs in MS1 (p<0.05) and REN (p<0.001) (Fig. 4D), supporting Gli factors as potent therapeutic targets. Comparable effects were observed in H28 (Fig. 4D), which was consistent with the idea that active upstream SHh signaling presented in the cell line. Overall, the pro-proliferation function strongly suggested Gli factors as potential therapeutic targets.

### Targeting Gli1 and Gli2 by a Novel Gli Inhibitor (Gli-I) has Better Effects than Targeting Smo (vismodegib) in Reduction of Cell Viability

**Figure 5:**
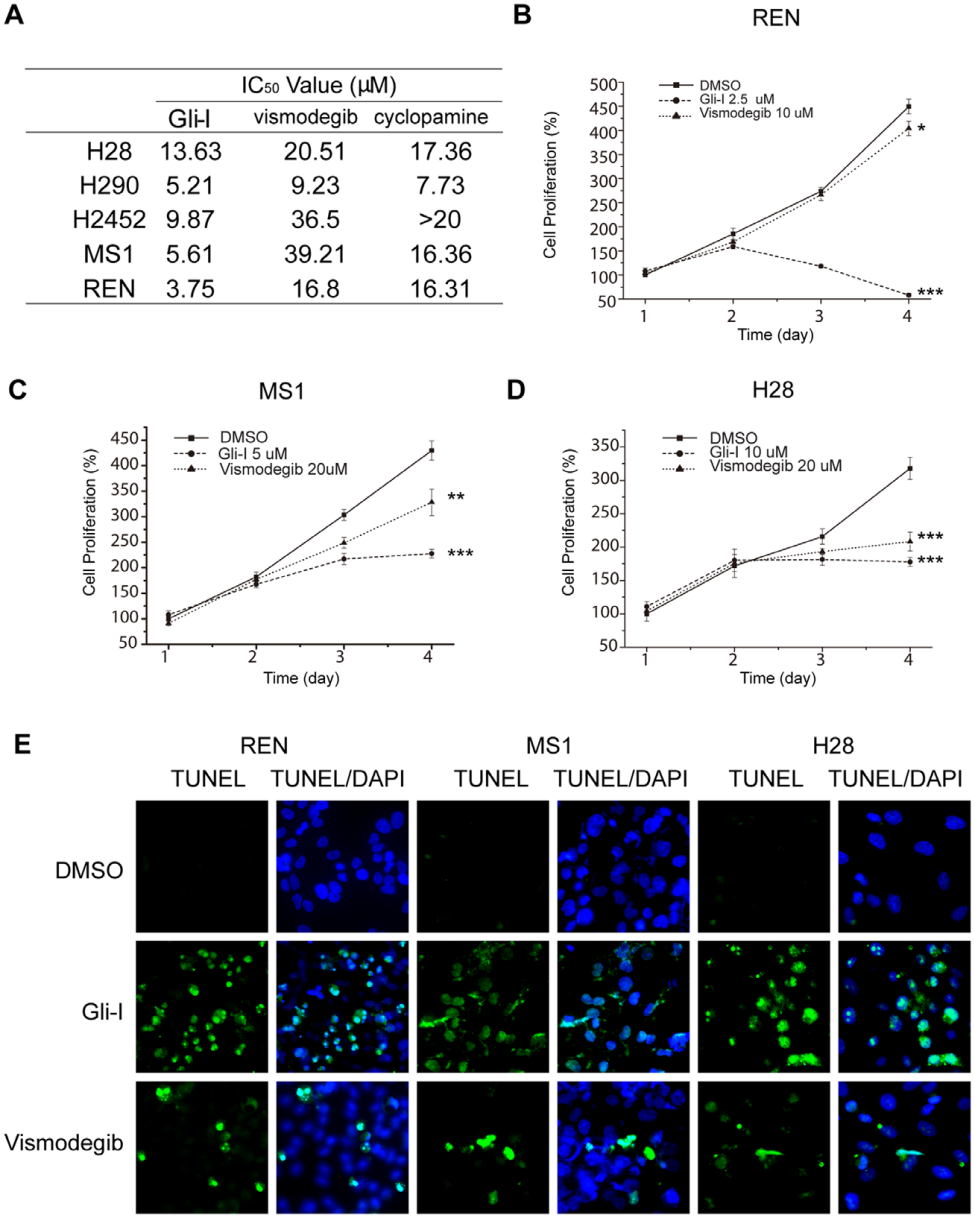
The Gli Inhibitor is More Potent than the Smo Inhibitors to Inhibit MPM Cell Proliferation. A , IC_50_ comparison of Gli-I and the Smo inhibitors. Cells were treated with corresponding compounds at 7 different concentrations for 72 hours to obtain a dose-respond curve in order to determine IC_50_ values. **B–D**, Gli-I achieved a better suppression of cell proliferation than the Smo inhibitor in three MPM cell lines. MTS assay was used to measure cell proliferation at 72 hours upon drug treatment. Two-sided student’s t-test was performed between DMSO and drug treated cells. A p value <0.05 was indicated as *, <0.01 as **, and <0.001 as ***. **E**, Apoptosis in Gli-I and vismodegib treated cells. TUNEL assays were performed at 40 hours after drug treatment, with TUNEL in green and DAPI in blue.

Our lab has developed a novel small molecule, the Gli inhibitor (Gli-I), which specifically inhibits Gli1 and Gli2 transcriptional activity, resulting in dramatic cytotoxicity in tumor cells that are Gli activity dependent [33]. We investigated the efficacy of Gli-I, and conducted a side-by-side comparison with two Smo inhibitors, vismodegib and cyclopamine, in MPM cell lines. Efficacy of Gli-I, vismodegib and cyclopamine were determined by cell proliferation assays in five MPM cell lines. The IC_50_ values of Gli-I ranged from 3.75 µM to 13.63 µM upon drug treatment for 72 hours, which were much lower than that of Smo inhibitors vismodegib and cyclopamine (Fig. 5A).

To confirm the cytotoxicity of Gli-I, we measured cell growth after Gli-I treatment. Cell proliferation was dramatically suppressed upon the treatment of Gli-I at concentrations lower than their corresponding IC_50_ values in different cell lines, i.e. 2.5 µM, 5 µM and 10 µM for REN, MS-1 and H28 respectively (Fig. 5B–D, p<0.001). In contrast, vismodegib showed no obvious effects when applied at the same concentration as Gli-I (data not shown), and had moderate effects in REN and MS-1 at 10 µM and 20 µM respectively, which were quadruple the concentration of Gli-I in the same assay (Fig. 5B, C). Vismodegib at 20 µM achieved comparable anti-proliferation effects as Gli-I at 10 µM in H28 (Fig. 5D). The comparison confirmed that H28 was sensitive to both Gli and Smo inhibitors, whereas REN and MS-1 was preferentially sensitive to Gli-I, which was consistent with the results of siRNA inhibition (Fig. 4). TUNEL assay was conducted in the cell lines upon 40 hr drug treatment at the same concentration as in the proliferation assay. Gli-I induced intensive apoptosis whereas vismodegib resulted in moderate apoptosis (Fig. 5E), which was consistent with the proliferation assay.

**Figure 6:**
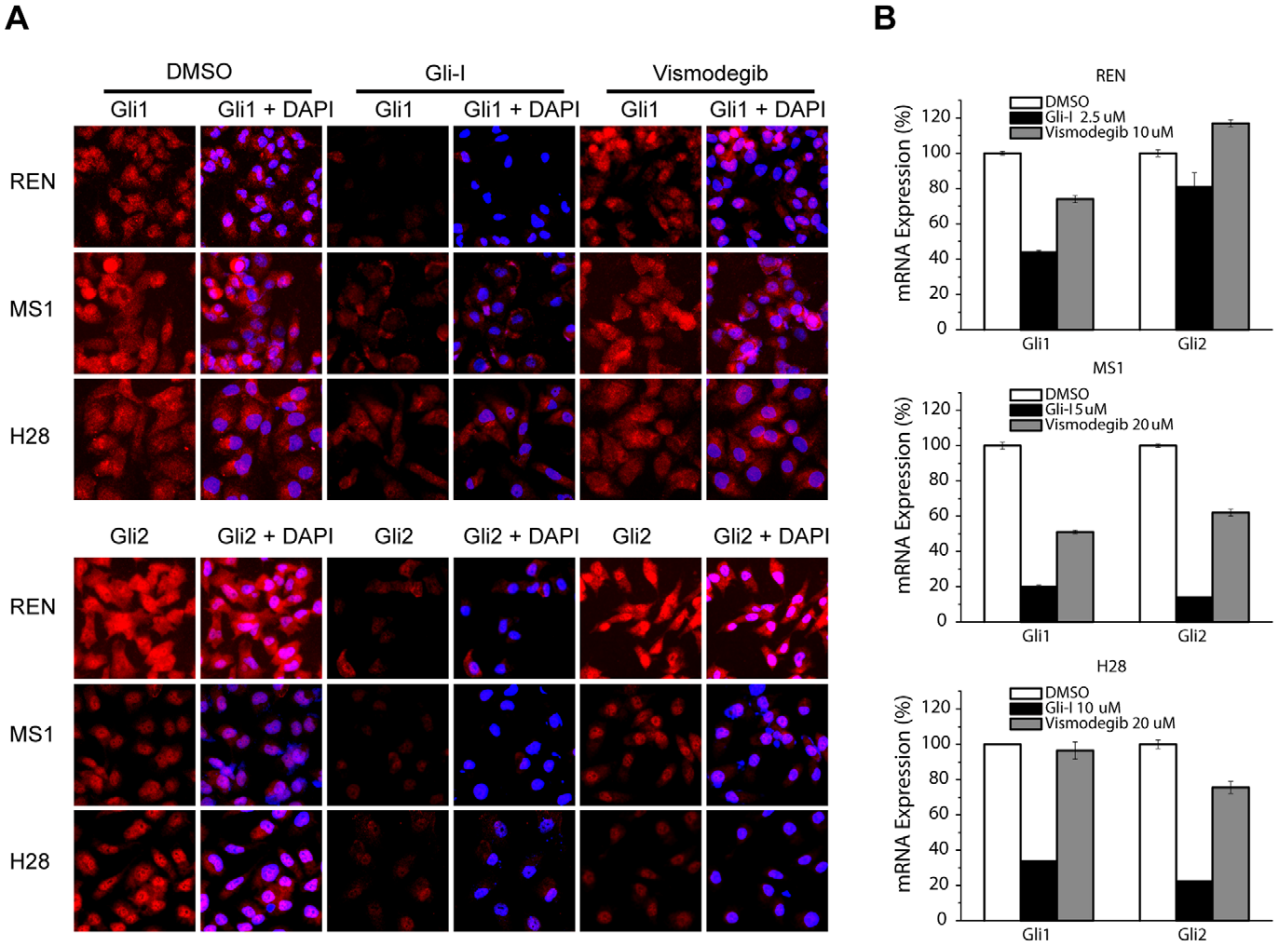
The Gli Inhibitor More Effectively Down-regulated Gli Expression than Vismodegib. A , Immunofluorescence staining of Gli1 and Gli2 upon 48 hr treatment of Gli-I and vismodegib in three MPM cell lines. **B**, RT-PCR analysis of *gli1* and *gli2* expression upon 30 hr treatment of Gli-I and vismodegib. The concentrations of both compounds for each cell line were the same in A and B, and were labeled in B.

To verify the specificity of Gli-I, protein expression of Gli1 and Gli2 were evaluated by immunofluorescence (IF) upon drug treatment for 48 hours (Fig. 6A). For REN and MS1, the down-regulation of Gli1 and Gli2 was significant by Gli-I, and only moderate by vismodegib; whereas for H28, the down-regulation were at comparable levels by the two compounds. RNA levels were monitored upon 30 hr treatment, and the suppression of *gli1* and *gli2* by Gli-I was more effective than that by vismodegib in all three cell lines (Fig. 6B).

Overall, the results strongly suggested that our novel compound Gli-I achieved a significant anti-proliferation effect by down-regulating Gli factors *in vitro*, and Gli factors might serve as more effective targets than Smo in treating MPM.

### Gli Inhibitor Inhibits Tumor Growth in a Xenograft Model

**Figure 7:**
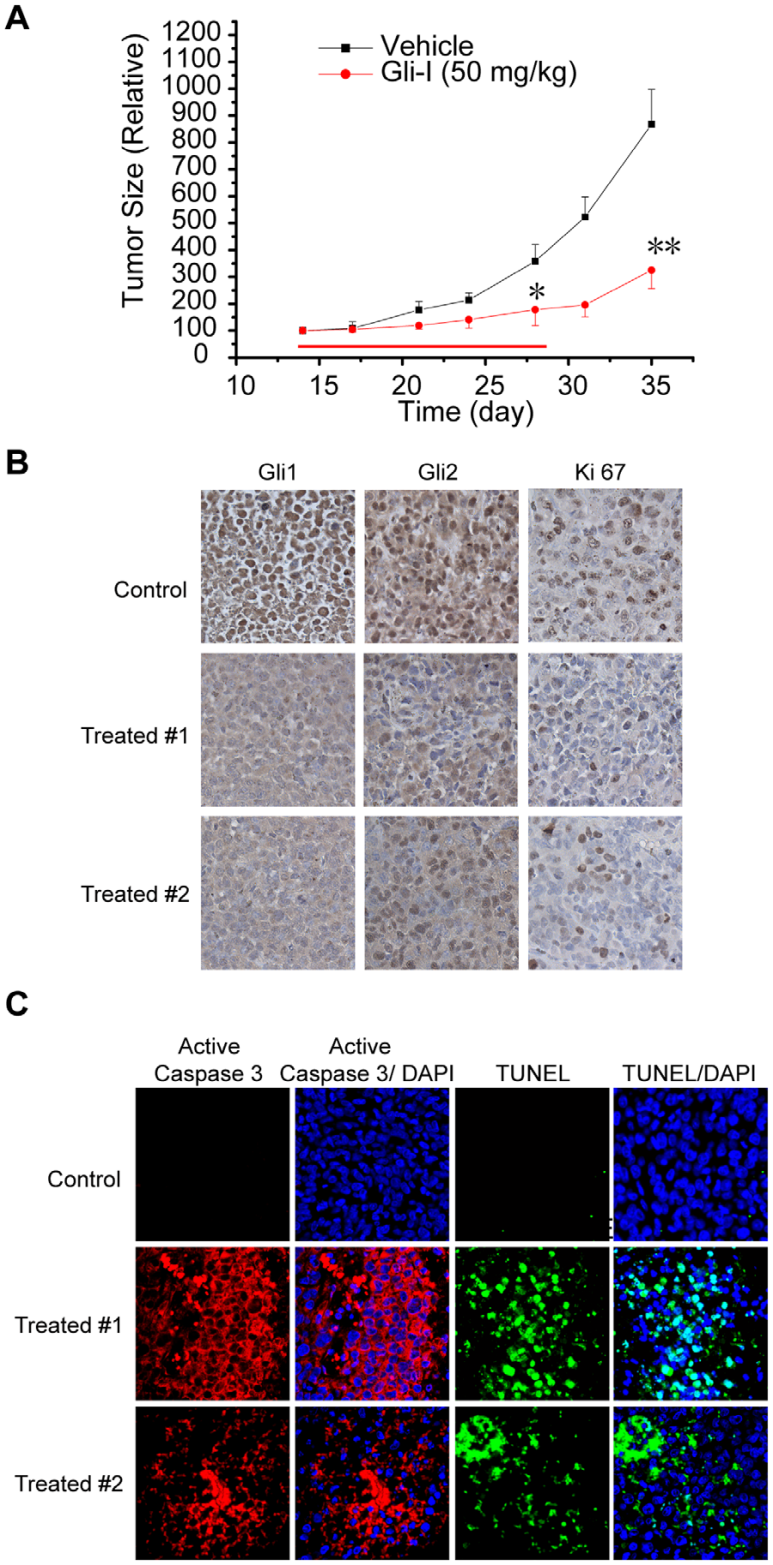
The Gli Inhibitor Suppressed Tumor Growth *in vivo* by Inhibiting Gli Proteins. A , MS-1 xenograft tumor growth upon Gli-I treatment. Tumor size was measured every 3–4 days. Red arrow indicated the period of drug treatment. Tumor volume was calculated by using the equation x^2^y/2 (where x<y), and presented as percentage of its initial volume on day 14. Two-sided student’s t-test was performed between control and drug treated mice at day 28 and day 35. A p value <0.05 was indicated as *and <0.01 as **. **B**, Gli1, Gli2 and Ki67 expression in the resected xenograft tumors by IHC. **C**, Apoptotic cells in the resected xenograft tumors by IF. Active Caspase 3 (red) with DAPI (blue) in two left columns, and TUNEL (green) with DAPI in two right columns.

To determine whether Gli inhibition represented a potent approach to suppress mesothelioma cell growth and tumorigenesis *in vivo*, the efficacy of Gli-I was examined in a MS-1 xenograft model. Fourteen days after implantation, when most tumors reached 100 mm^3^, mice started to receive daily IP injections of Gli-I at 50 mg/kg for 14 days. Gli-I treatment significantly inhibited tumor growth by 51% at the end of the 14-day treatment compared with the control group (Fig. 7A, p<0.05). In addition, the xenograft re-growth after treatment withdrawal was monitored for another 7 days. The inhibition of tumor growth was sustained and more substantial (63%) compared with control group (Fig. 7A, p<0.01), indicating durable effects of Gli inhibition. No obvious change of body weight was observed during the course, or any noticeable toxicity in major organs from the treated mice (data not shown).

In addition, we examined the resected xenograft tumor specimens after the completion of the *in vivo* experiment (Fig. 7B). Gli1 and Gli2 proteins were down-regulated in Gli-I treated tumors compared with control, consistent with *in vitro* results (Fig. 7B). The decrease of Ki-67 levels confirmed the reduction of proliferation in Gli-I treated tumors (Fig. 7B). Dramatically increased apoptosis was visualized by active caspase 3 staining and TUNEL assays (Fig. 7C). The xenograft study strongly suggested that Gli-I had an extensive anti-tumor efficacy *in vivo* by inhibiting Gli factors.

### Gli-I has Synergistic Effects with Vismodegib and Pemetrexed Disodium

**Figure 8:**
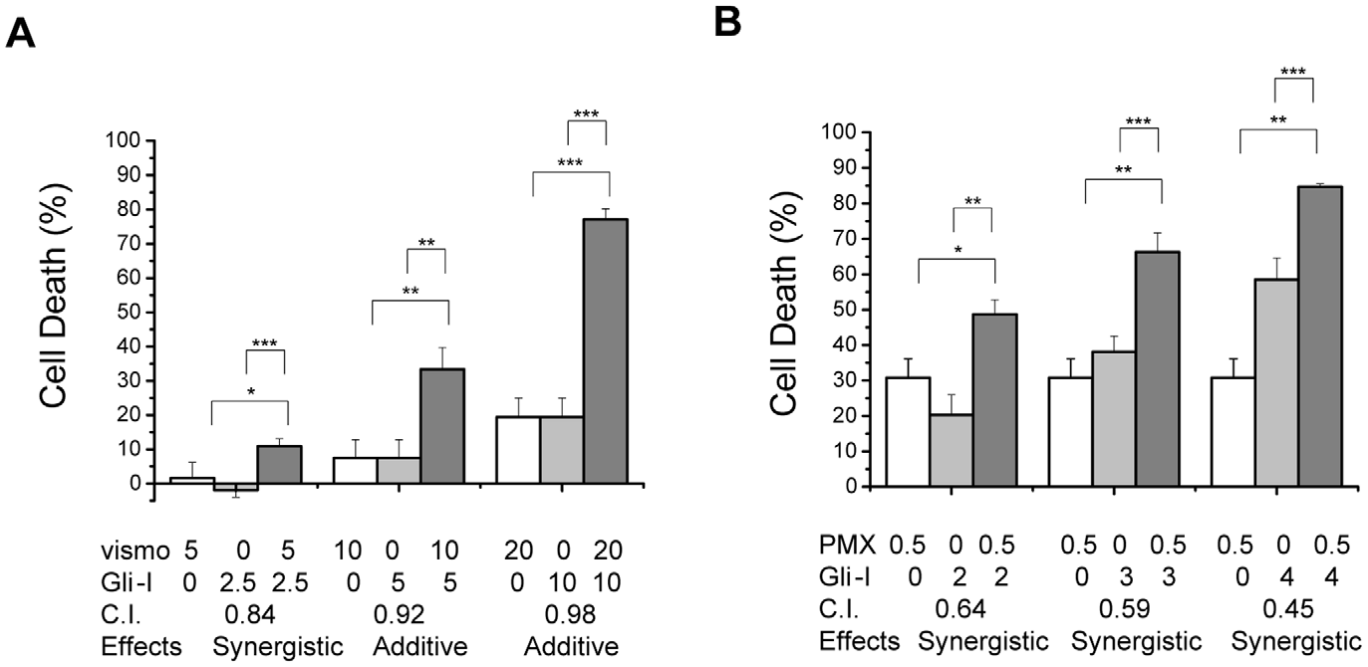
Synergistic Effects of the Gli Inhibitor with Vismodegib and Pemetrexed. A-B , Cells were treated with Gli-I, vismodegib and pemetrexed at the concentration indicated in the figure. Cell survival was measured by MTS assays at 72 hours of drug treatment. The combinational effects were further quantified using the Chou-Talalay Method to obtain the Combinational Index (CI), where CI <1, = 1, >1 represent synergism, additive effect, and antagonism respectively. Two-sided student’s t-test was performed between single and double treatment. A p value <0.05 was indicated as *, 0.01 as **, and 0.001 as ***.

To further characterize the anti-tumor efficacy of Gli-I, we examined the combinational treatments of Gli-I plus vismodegib or the chemotherapy drug pemetrexed. We hypothesized that Gli-I might show a synergistic effect with vismodegib, as the latter may further suppress Gli activation in cells with active SHh/Smo signaling. In H28 where SHh-dependent Gli activation exists, the combinational treatment of Gli-I and vismodegib led to a significantly better cytotoxicity than either single treatment (Fig. 8A). The combinational effects were further quantified using the Chou-Talalay Method to obtain the Combinational Index (CI), where CI <1, = 1, >1 represent synergism, additive effect, and antagonism respectively. The CI analysis showed synergistic effects at lower dose, and additive effects at higher dose.

We also examined if Gli-I and pemetrexed might show synergistic effects. Gli activation has been suggested to maintain the stem cell population, which contributes to drug resistance and recurrence [34]. Therefore, it is possible that Gli-inhibition might improve the effectiveness of pemetrexed. Pemetrexed is one of the most commonly used first line chemotherapy drugs. Improvement of penetrexed efficacy would be beneficial to MPM patients. We observed a dramatic synergistic effect when Gli-I was applied together with pemetrexed (Fig. 8B).

Both studies suggested the combinational treatments as meaningful approaches to treat MPM, further supporting Gli factors as promising therapeutic targets in MPM.

## Discussion

Aberrant Gli activation has been implicated in tumorigenesis in a wide variety of tumors [22]. The current study reported the essential role of the Gli family of transcriptional factors for MPM tumor growth. First, a large majority of MPM tissues had significantly higher expression of Gli and their downstream transcriptional targets (Fig. 1). Secondly, significant suppression of MPM cell proliferation *in vitro* was achieved by inhibiting Gli by siRNAs (Fig. 4). Furthermore, the application of a novel Gli inhibitor (Gli-I), which specifically inhibited Gli factors, led to a dramatic reduction of MPM cell proliferation both *in vitro* and *in vivo* (Fig. 5, 6, 7). All the evidence highlighted Gli as a potent therapeutic target in MPM.

Our results showed that directly targeting Gli1 and Gli2 achieved greater cytotoxicity than targeting Smo in MPM cells. Inhibition of Gli by siRNAs was more effective than that of Smo to suppress cell proliferation (Fig. 4). In addition, Gli-I was more potent to reduce cell growth than two Smo inhibitors, vismodegib and cyclopamine (Fig. 5). Furthermore, Gli-I reduced Gli levels more effectively than vismodegib (Fig. 6). In fact, the reduction of Gli levels by vismodegib was moderate even at a much higher concentration (2–4 fold higher than Gli-I).

What are the possible explanations for the difference in effectiveness of Smo and Gli inhibition? It is possible that inhibition of an upstream factor such as Smo might not effectively translate into the reduction of downstream Gli factors. However, we favored the explanation that the better efficacy was likely due to the Hh-independent Gli activation in MPM. In other words, Smo inhibition had little effect on Hh-independent Gli activation, and thereby was less potent in growth inhibition of cancer cells lacking upstream canonical Hh/Smo signaling. The coexistence of Hh-dependent and-independent Gli activation has been documented by a growing body of evidence in a variety of cancers, and the underlying mechanisms of Hh-independent Gli activation involved multiple signaling pathways, such as TGFβ, EGFR, and RAS and AKT/PI3K pathways [9], [22]. It is likely that MPM employs both Hh-dependent and -independent Gli activation in a context dependent manner. Our findings of elevated Smo and SHh expression in some tissues, as well as suppression of cell proliferation through the inhibition of Smo via siRNA and small molecule inhibitors, suggested that the conventional Hh pathway plays a role in MPM. However, the lack of correlation between SHh and Gli expression in MPM patient tissues and cultured cell lines indicated that the upstream signals from SHh through Smo might not be the only stimuli of aberrant Gli activation. Consistently, we observed differentiated responses to Smo and Gli inhibition in the three MPM cell lines. It is possible that H28, which had a comparable response to Smo and Gli inhibition, relied on the Hh-dependent Gli activation, whereas REN and MS1, which preferentially responded to Gli inhibition, harbored the Hh-independent Gli activation to a significant extent. The molecular mechanism underlying potential non-canonical Gli activation in MPM were currently under investigation in our group, and might further support Gli as a potent therapeutic target to treat MPM.

A very recent publication by Shi *et.al* documented the essential role of Hh signaling in MPM, and suggested Smo inhibition as a therapeutic approach [35]. Our study provided a different perspective to understand the molecular mechanism of MPM, and suggested Gli inhibition as a promising approach to treat MPM patients. We reported different expression profiles of the Hh pathway components, for example, SHh, Gli1 and Gli2. The difference is possibly due to the small patient numbers, and the limited normal pleura samples that were used as the expression baseline. However, we suspected that the existence of the Hh-independent Gli activation might complicate the overall expression profiles and thereby contribute to the different expression profiles reported by the two studies. Moreover, Shi *et.al* documented MPM cell lines that were insensitive to Smo inhibition. It would be interesting to examine if Hh-independent Gli activation plays a role in these cell lines and if these cell lines respond to Gli inhibition.

Moreover, both *in vivo* and *in vitro* studies strongly suggested that the novel Gli inhibitor, Gli-I, held a great potential to be a potent therapeutic agent to suppress tumor growth. Our group has accumulated a large body of evidence suggesting that the small molecule specifically and effectively inhibited Gli1 and Gli2 both *in vivo* and *in vitro*, dramatically suppressed tumor growth in several tumor types, such as lung cancer, melanoma and MPM as reported here, and had a very low toxicity profile *in vivo*. The synergistic effects of Gli-I with other therapeutic compounds also indicated the potential benefits to further develop the novel Gli inhibitor.

In summary, the Gli family of transcriptional factors plays a critical role in MPM. Inhibition of Gli is highly effective at suppressing cell proliferation in MPM cells in contrast to targeting the upstream factor Smo. Our small molecule Gli inhibitor, Gli-I, has a strong potential to become a novel, clinically effective approach to treat MPM.

## Supporting Information

Figure S1 **Correlation between Smoothened and Gli.** Expression of *smo* and *gli1 gli2* was quantified by qPCR. The expression of *smo* was plotted against *gli1* (upper) and *gli2* (lower).
(TIF)
