# Supplementary figures and images for: Gli as a Novel Therapeutic Target in Malignant Pleural Mesothelioma
Source: PLoS One. 2013 Mar 6;8(3):e57346. doi: 10.1371/journal.pone.0057346 (PMC3590216; doi:10.1371/journal.pone.0057346)

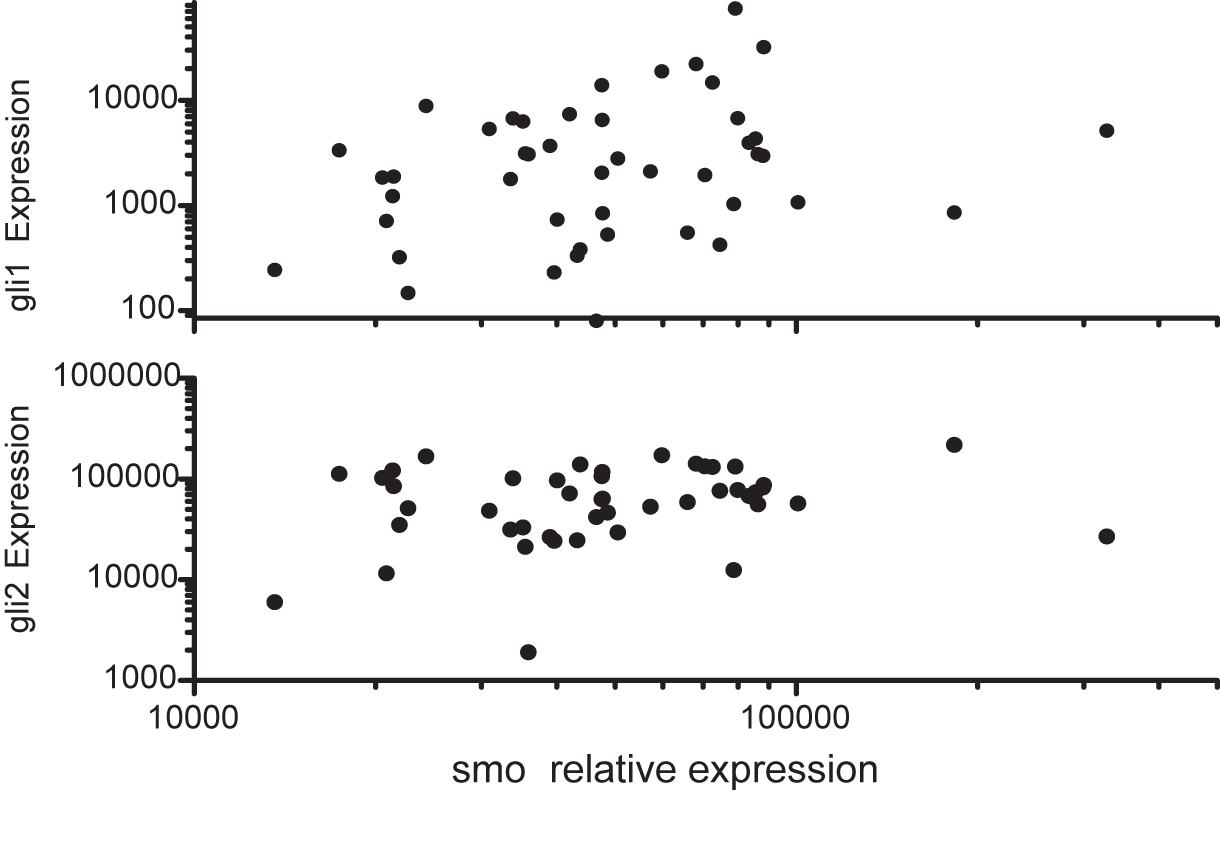

Supplement: Figure S1 — Correlation between Smoothened and Gli. Expression of smo and gli1 gli2 was quantified by qPCR. The expression of smo was plotted against gli1 (upper) and gli2 (lower). (TIF) [file pone.0057346.s001.tif]
